# Tremor in Idiopathic Cervical Dystonia – Possible Implications for Botulinum Toxin Treatment Considering the Col-Cap Classification

**DOI:** 10.5334/tohm.63

**Published:** 2020-07-07

**Authors:** Sanjay Pandey, Alexandre Kreisler, Artur Drużdż, Bo Biering-Sørensen, Jaroslaw Sławek, Laurent Tatu, Wolfgang H. Jost

**Affiliations:** 1 Department of Neurology, Govind Ballabh Pant Institute of Postgraduate medical education and research, JLN Marg, New Delhi, IN; 2 Service de Neurologie et Pathologie du Mouvement, CHRU de Lille, Lille, FR; 3 Department of Neurology, Municipal Hospital, Poznan, PL; 4 Movement Disorder Clinic, Department of Neurology, Rigshospitalet, Copenhagen, DK; 5 Department of Neurological-Psychiatric Nursing, Medical University of Gdańsk, Gdańsk, PL; 6 Department of Neurology, St. Adalbert Hospital, Gdańsk, PL; 7 Department of Neuromuscular diseases and Department of Anatomy, CHRU Besançon, University of Franche-Comté, Besançon, FR; 8 Department of Anatomy, CHRU Besancon, University of Franche-Comté, Besançon, FR; 9 Parkinson-Klinik Ortenau, Kreuzbergstr, Wolfach, DE

**Keywords:** Cervical dystonia, Tremor, Torticaput, Botulinum toxin, Ultrasonography

## Abstract

**Background:**

Tremor is an important phenotypic feature of dystonia. Using the new concept (Col-Cap) of classification we examined the frequency of tremor in cervical dystonia (CD) patients, their main subtypes and muscles injected.

**Methods:**

In this large study conducted at multiple movement disorder centres in Europe and India, between January and June 2019, we examined 293 patients with idiopathic CD who were all treated with botulinum toxin (BTX).

**Results:**

The dystonic head tremor (DHT+) was present in 57.6 % of CD patients and they had a significantly longer duration of symptoms than patients without head tremor (DHT–). In DHT+ patients torticaput was the most common subtype and the majority (63.3%) had one or two subtypes only. There was no significant difference between the number of unilateral injections for any of the muscles in the DHT+ and DHT– groups, while the number of patients receiving bilateral injections in splenius capitis (78 vs 25; p = 0.00001), sternocleidomastoid (31 vs 6; p = 0.0005), trapezius (28 vs 9; p = 0.01), and obliquus capitis inferior (15 vs 2; p = 0.008) were significantly more in the DHT+ group. The mean doses of all three types of BTX/A were not significantly different between the two groups.

**Conclusions:**

The frequency of head tremor was 57.6% in our CD patients and torticaput was the most common dystonic subtype associated with tremor. Simple forms of CD seemed more likely associated with head tremor, than complex forms of CD. Most of the DHT+ patients received bilateral injections. The use of ‘Col-Cap’ classification was helpful in the identification of muscles likely to be involved in tremor in CD patients.

## Introduction

Tremor is an important motor phenotype of dystonia, observed in 14 to 86.7% of the patients [1]. Most studies have reported a greater occurrence of tremor in cervical dystonia (CD) than dystonia involving other body regions [1]. The selection of muscles to be injected in CD patients is very individual and there are no fixed guidelines or recommendations regarding the doses [2,3]. Splenius capitis (SCap) and sternocleidomastoid (SCM) are commonly injected for dystonic head tremor, especially by physicians who do not use ultrasonography (USG). However, this approach is more empiric than evidence-based [4]. Some recent studies have suggested that injecting obliquus capitis inferior (OCI) may be more effective in such patients [5]. A new concept (Col-Cap) was introduced in 2009 to broaden the number of CD patterns and to increase the effect of the treatment [6]. By the introduction of this (Col-Cap) concept, a distinction is made between the respective caput and collis type, for example, between torticaput and torticollis [7]. In torticaput, muscles that induce a rotation rostral to C3 are dystonic and the head demonstrates a pivotal movement in relation to the neck. In torticollis, movement takes place caudal to C2, and rotation of the neck occurs in relation to the trunk [7]. The present study, conducted at multiple centres in Europe and India, aimed to examine the frequency of tremor in CD patients in a large cohort of patients, to determine the main subtypes associated with dystonic head tremor and to precise the strategy of injection in daily practice. Our study can potentially be helpful in guiding the muscle selection in CD patients with head tremor.

## Patients and methods

At seven specialized movement disorders centres we prospectively examined 311 consecutive patients with CD. Patients were included if they had idiopathic CD, with pronounced symptoms, interfering with their daily activities, admitted at least three months after their previous botulinum toxin type A (BTX/A) treatment and whose effect had worn off. Eighteen patients were excluded for incomplete data collection thus resulting in a sample size of n = 293. The centers involved included Besançon (France), Copenhagen (Denmark), Gdańsk (Poland), Lille (France), New Delhi (India), Poznań (Poland), and Wolfach (Germany). All investigators are specialists in movement disorders and have long-term experience with BTX/A and CD treatment. All injections were performed with the use of USG guidance and sometimes in combination with electromyography (EMG). We used the “Col-Cap” classification to identify the CD pattern and muscles to inject. The doses were chosen individually, and there were no differences between the centers. Depending on the national legal situation, the study was approved by the responsible ethics committees.

## Statistical analysis

The data were entered in the Microsoft office excel sheet and analysed using the SPSS software version 18. Values were expressed as mean ± standard deviation and as percentages and ranges. Intergroup comparisons were performed using the chi-squared test. The threshold for statistical significance was set to *p* < 0.05.

## Results: (Tables 1 and 2)

**Table 1:** Demographic data and main subtypes of idiopathic cervical dystonia patients with (DHT+) and without (DHT–) dystonic head tremor.

	DHT+	DHT–	*p* value

Total number of patients	169 (57.6%)	124 (42.3%)	–
Age [Mean ± standard deviation (SD), Range] years	56.2 ± 13.11, 23–90	54.2 ± 12.61, 29–74	0.19
Sex (Male: M; Female: F)	F: 120 (71.0 %) ; M: 49 (29.0 %)	F: 77 (62.1 %); M: 47 (37.9 %)	
Duration of illness (Mean ± SD, Range) in years	11.8 ± 8.69, 0.5–39	9.5 ± 8.91, 0.6–51	**0.02**
**Number of subtypes**			
One subtype	35 (20.7%)	14 (11.2%)	**0.03**
Two subtypes	72 (42.6%)	49 (39.5%)	0.59
Three subtypes	37 (21.8%)	32 (25.8%)	0.43
Four subtypes	20 (11.8%)	21 (16.9%)	0.21
Five subtypes	05 (02.9%)	06 (04.8%)	0.40
Six subtypes	00	02 (01.6%)	–
**Main subtype**			
Torticaput	97 (57.3%)	50 (40.3%)	**0.01**
Laterocaput	22 (13.0%)	25 (20.1%)	0.09
Laterocollis	18 (10.6%)	12 (9.6%)	0.78
Torticollis	12 (7.1%)	14(11.2%)	0.21
Retrocaput	7 (4.1%)	7 (5.6%)	0.55
lateral shift	5 (2.9%)	00	0.19
Retrocollis	4 (2.3%)	4 (3.2%)	0.65
Anterocollis	2 (1.1%)	6 (4.8%)	0.05
Anterocaput	1 (.05%)	4 (3.2%)	0.08
Anterior sagittal shift	1 (.05%)	1 (.05%)	0.82

**Table 2:** Details of the muscles injected and the doses of botulinum toxin in idiopathic cervical dystonia patients with (DHT+) and without (DHT–) dystonic head tremor.

Name of the muscle	DHT+ group (n = 169)	DHT– group (n = 124)	*p* value

Sternocleidomastoid unilateral	110 (65.0%)	87 (70.1%)	0.36
Sternocleidomastoid bilateral	31 (18.3%)	06 (04.8%)	**0.0005**
Splenius capitis unilateral	76 (44.9%)	66 (53.2%)	0.16
Splenius capitis bilateral	78 (46.1%)	25 (20.1%)	**0.00001**
Trapezius unilateral	62 (36.6%)	68 (54.8%)	0.056
Trapezius bilateral	28 (16.5%)	09 (07.2%)	**0.01**
Levator scapulae unilateral	61 (36.0%)	54 (43.5%)	0.19
Levator scapulae bilateral	13 (07.6%)	13 (10.4%)	0.40
Semispinalis capitis unilateral	60 (35.5%)	37 (29.8%)	0.30
Semispinalis capitis bilateral	06 (03.5%)	10 (08.0%)	0.09
Obliquus capitis inferior unilateral	45 (26.6%)	41 (33.0%)	0.23
Obliquus capitis inferior bilateral	15 (08.8%)	02 (01.6%)	**0.008**
Semispinalis cervicis unilateral	27 (15.9%)	26 (20.9%)	0.27
Semispinalis cervicis bilateral	07 (04.1%)	05 (04.0%)	0.96
Longus colli unilateral	24 (46.1%)	23 (18.5%)	0.31
Long colli bilateral	02 (01.1%)	0	–
Scalenus medius unilateral	12 (07.1%)	12 (09.6%)	0.42
Scalenus medius bilateral	04 (02.3%)	06 (04.8%)	0.24
Splenius cervicis unilateral	10 (05.9%)	10 (08.0%)	0.47
Splenius cervicis bilateral	0	0	–
Total	855	576	0.51
**Total dosages of botulinum toxin**			
OnabotulinumtoxinA [Mean ± standard deviation (SD), Range]	99 (162.5 ± 68.5, 30–300) units	53 (156.0 ± 49.3, 50–240) units	0.54
IncobotulinumtoxinA (Mean ± SD, Range)	26 (175.6 ± 123.5, 50–590) units	21 (155.8 ± 63.5, 48–270) units	0.50
AbobotulinumtoxinA (Mean ± SD, Range)	44 (640.2 ± 216.0, 102.5–1020) units	50 (654.5 ± 358.4, 170–1520) units	0.81

Out of 293 patients of CD, dystonic head tremor was present (DHT+) in 57.6 % (n = 169) and absent (DHT–) in 42.3% (n = 124) of the patients (Table 1). In the DHT+ group, 29% of the patients and in the DHT– group 37.9% of the patients were males. The duration of symptoms was longer in the DHT+ group than in the DHT– group (11.8 ± 8.6 versus 9.5 ± 8.9 years; p = 0.02). More patients had only one dystonic subtype in the DHT+ group, (35/169) than in the DHT– group (14/114) (p = 0.03). The majority (63.3%) of the DHT+ patients had one (20.7%) or two (42.60%) subtypes only (Table 1). Two patients in DHT– group had six subtypes compared to none in the DHT+ group. Torticaput (most common dystonic subtype) was more frequent in the DHT+ group with 57.3% than in the DHT– group (40.3%) (p = .01). The other major subtypes in DHT+ patients were laterocaput (n = 22; 13.0%), laterocollis (n = 18; 10.6%), and torticollis (n = 12; 7.1%). The other main subtypes present in DHT– group were laterocaput (n = 25; 20.2%), torticollis (n = 14; 11.3%), and laterocollis (n = 12; 9.67%). Antecollis was more frequent in the DHT– group (n = 6; 4.8%) than the DHT+ group (n = 2; 1.2%), but the difference did not reach significance (p = 0.05). Most of the patients received multiple cycles of BTX injection in the past: 167/169 in the DHT+ group and 119/124 in the DHT– group. Unilateral injection of the SCM muscle was most frequently performed in both the DHT+ (n = 110) and DHT– groups (n = 87) (p = 0.36) (Table 2). Overall there was no significant difference between the number of unilateral injection for any of the muscles in the DHT+ and DHT– groups. In the DHT+ group, bilateral injections in the SCap, SCM, Trap and OCI muscles were more frequent than in the DHT– group.

The mean ± SD doses of onabotulinumtoxinA (162.52 ± 68.58 units versus 156.02 ± 49.39; p = 0.54), incobotulinumtoxinA (175.62 ± 123.57 versus 155.85 ± 63.58 units; p = 0.50), and abobotulinumtoxinA (640.28 ± 216.05 versus 654.50 ± 358.47 units; p = 0.54) were not significantly different between the two groups (Table 2).

## Discussion

The current definition of dystonia includes tremor as one of the important motor phenotype. Table 3 summarizes literature data dedicated to the frequency of tremor in dystonia [1,8,9,10,11,12,13,14,15,16,17,18,19]. Most studies have reported a greater occurrence of tremor in CD than any other focal dystonia and the findings of our study from a large sample size further strengthens this observation [1]. According to literature, tremor is more likely to be associated with the longer duration of CD. In our study DHT+ patients actually had a longer disease duration than DHT– patients (11.8 ± 8.6 versus 9.5 ± 8.9 years; p = 0.02).

**Table 3:** Studies reporting frequency of tremor in idiopathic cervical dystonia patients.

Author/year		No of patients	Number of cervical dystonia patients	Frequency of head tremor

Huizdosova et al [8]	2020	120	Cervical dystonia	58.3 % (n = 70)
Merola et al [9]	2019	1608	Cervical dystonia	18.1% (n = 291)
Gigante et al [10]	2015	173	72 cervical dystonia patients	Overall 34% (n = 59) had tremor [12 patients- head tremor 34 patients-arm tremor 13 patients-both sites]
Pandey et al [11]	2017	90	Adult onset isolated dystonia Cervical dystonia-41 Limb dystonia-34 Cranial dystonia-15	Overall 45.5% (n = 41) Cervical dystonia-21(51.2%)
Erro et al [12]	2013	473	Adult-onset primary dystonia patients including 207 patients with cervical dystonia	Overall 55.4% (n = 262) had tremor; 196 patients (41.4%) had head tremor
Defazio et al [13]	2013	429	Primary adult onset dystonia patients including 118 cervical dystonia patients	22/34 (64.7) patients with tremor had cervical dystonia
Pal et al [14]	2000	114	Cervical dystonia	68.4% (n = 78) had head tremor and 34.6% of them (27) had tremor as one of the first symptoms
Deuschl et al [15]	1997	55	Idiopathic spasmodic torticollis	60%
Chan et al [16]	1991	266	Idiopathic cervical dystonia	28%
Jankovic et al [17]	1991	300	Cervical dystonia	71%
Couch et al [18]	1976	30	Torticollis	26 (86.6%)
Marsden et al [19]	1971	42	Idiopathic torsional dystonia	6 (14%)

The involved muscles in CD can be classified into three groups: dystonic, antagonist and compensatory [6]. The “antagonist muscles” are passively tensed by abnormal movements or posture and sometimes induce opposite movements or tremor [6]. The SCM and SCap are the best examples of the antagonist muscles frequently involved in torticaput subtype. In our study torticaput was significantly more common in DHT+ patients; this may be explained by more involvement of the antagonist muscles in this subtype. On the other side, anterocaput and anterocollis were less associated with DHT+, maybe due to the lack of involvement of antagonist muscles. Godeiro-Junior et al retrospectively evaluated medical charts of CD patients and classified them into two groups: with (HT+) and without (HT–) tremor. They reported head tremor in 38.2% of patients [20]. The most common type of CD present in HT+ patient was the combination of torticollis+laterocollis (26.6%) followed by torticollis+laterocollis+anterocollis (20%), torticollis+retrocollis (15.6%), torticollis (13.4%), and torticollis+laterocollis+retrocollis (11.1%). Their observations were different from our study where the majority (>62%) of the patients had a simple type of CD (one or two subtypes). Godeiro-Junior et al also observed that the HT+ patients had a better response to BTX/A in the long term compared to HT–, but they did not provide any explanation for that [20]. This observation is interesting and requires further validation considering that the clinical experience of our group is different.

Pal et al prospectively evaluated 114 consecutive patients with CD and classified them into two groups: with (HT+) and without (HT–) tremor, but they did not evaluate the response to BTX/A [14]. Seventy-eight patients (68.4%) had head tremor (HT+) while 36 patients (31.5%) did not. Some dystonic movements were more frequent in the HT+ group than in the HT– group: head rotation (HT+: 88.5%, HT–: 69.4%) and antecollis (HT+:12.8%, HT–: 5.5%). Godeiro-Junior et al and Pal et al studies are not directly comparable to the present work, as they did not use the “Col-Cap” classification. In this point of view, the “Col-Cap” classification may be used to standardize the assessment of the CD patients.

There is no established protocol for selecting muscles for BTX/A injection. But, there are some important considerations regarding the selection of muscles including the abnormal posture, muscle hypertrophy, pain, muscle palpation and presence of tremor [4,21]. Misra et al reported real-life data on response to BTX in CD collected in 404 adult patients from 38 centers across 10 countries [22]. The clinical assessment of the subjects also included the presence of tremor using the Tsui tremor subscale [23]. Overall, data from this study revealed that more subjects responded among subgroups presenting without baseline tremor (33.2% vs 23.7% with tremor) [22]. The authors concluded that factors most strongly associated with responses were age and absence of baseline head tremor (OR 1.5; not significant) [22]. This observation regarding the relationship between the tremor and response to BTX may have clinical implications for improvement, but the results of different studies are inconsistent [4].

In CD patients the correct identification of neck muscles involved in dystonic head tremor may occasionally be hampered by spontaneously changing muscular activation patterns. To overcome these difficulties, USG is mainly used to inject muscles with good accuracy, and EMG is mainly used to select muscles to be injected [5]. Schramm and colleagues recruited 35 CD patients with dystonic head tremor and they performed USG guided EMG recordings from the SCap and OCI muscles [5]. They also assessed the depth and thickness of the SCap, semispinalis capitis (SsCap) and OCI muscles. They documented bursts like tremor activity present in bilateral OCI in 25 (71.4%) and unilateral OCI in 10 (28.5%) patients. Tremor activity was present in one SCaP in 18 (51.4%) patients and bilateral SCaP in 2 (5.7%) patients. Bilateral OCI and ipsilateral SCaP were the most active muscles present in 11 (31.4%) patients. The authors concluded that in tremulous CD, muscular activity is commonly present in OCI muscles and targeted BTX injection into this deep muscle will likely improve the outcome. In our study, USG was used in all the patients, but the EMG was not used for all. Our results also indicate that a large number of our patients with head tremor received bilateral injections in the SCaP (n = 78; 46.2%), SCM (n = 31; 18.3%), Trap (n = 28; 16.6%) and OCI (n = 15; 8.9%) muscles.

Schramm et al also observed that the depth and thickness of the OCI, SCaP and SsCap muscles were variable on USG. However, the validity of USG on the measurement of cervical muscles is still under debate [24]. When the muscle is contracted, the size and thickness, of course, change a lot, which is a problem in patients with CD.

Jankovic et al reported in an open trial of BTX the treatment of 51 patients with disabling dystonic tremor including 42 patients with head tremor who were injected in bilateral SCap and one or both SCM muscles [25]. An average of 242 ± 75 units of OnabotulinumtoxinA was injected per visit and the response was better in dystonic head tremor patients. In our study, the mean total doses injected were lower, but at the same time, more muscles were injected in our DHT+ patients compared to the patients in the study by Jankovic et al.

Wissel and colleagues treated 43 patients with head tremor with BTX injections in neck muscles [26]. Twenty-nine patients were classified as tremulous cervical dystonia (TCD) and 14 patients were classified as tremor without dystonia (HT). The mean total dose received in the TCD group was higher [500 abobotulinumtoxinA units (Range 320–720 U)] than in the HT group [400 U (160–560 U)]. All patients with HT and 26 of the 29 patients with TCD showed subjective improvement in their head tremor. All HT patients received an injection in both SCap muscles, whereas the majority of the TCD patients received injections in SCap, SCM, Trap, levator scapulae and semispinalis cervicis. In the present study, most of the patients received bilateral injections in conventional muscles such as SCap and SCM as well as in “new” muscles such as OCI. The doses of BTX/A for all three types of preparations (Ona, inco and abobotulinumtoxinA) did not differ between the DHT+ and DHT– patients.

There are some important limitations to our study. First, this was a prospective study primarily focussing on different subtypes of CD using ‘Col-Cap’ classification. This aim was not to provide a detailed evaluation of tremor, therefore some information are lacking (such as the intensity and the subtype of the tremor: no-no, yes-yes or multidirectional). Moreover, we did not look for tremor in non-dystonic body segments including upper limbs (also labelled as tremor associated with dystonia). Second, EMG guided injections were not performed in all the centres, so we are not able to comment on the EMG activity in different muscles. Third, we did not have any follow-up data to demonstrate the improvement of tremor in our CD cohort following the BTX/A injection. Fourth, the majority of our patients had received multiple sessions of BTX/A injection in the past, that might have influenced the muscle selection and presence of tremor.

Despite all these limitations, our study has provided some important data regarding tremor in CD patients. First, the frequency of tremor in our cohort was 57.6%, which is similar (Table 3) to the other studies highlighting that among all the focal dystonia patients tremor is most commonly seen in CD patients. Second, our CD patients with tremor had a longer duration of symptoms in comparison with patients without tremor. Our finding is consistent with the current literature showing that tremor in dystonia is more likely to be seen as dystonia progresses. Also, there are likely to be two groups of patients: either the tremor is present at the onset, or the tremor is seen after many years. However, if tremor during follow up is related to CD evolution or BTX/A treatment remains unknown. Only a long-term prospective study in de novo patients may provide an answer. Third, our study has revealed that torticaput is more likely to be associated with tremor compared to torticollis. As the majority of DHT+ patients presented only with one or two subtypes, we can hypothesize that simple forms of CD are more likely to be associated with head tremor, than complex forms of CD (three or more subtypes). Therefore, the choice of muscles selected for injection may be better determined by the ‘Col-Cap’ classification. Fourth, more patients received bilateral injections in the SCM, SCap, OCI and Trap in the DHT+ group than in the DHT– group. Bilateral OCI has been considered to be involved in the pathogenesis of tremor in CD patients, however, the involvement of other muscles may also be important in the treatment of CD patients with tremor. Fifth, the injected doses of BTX/A were not different between our two groups, however, more muscles were injected in the DHT+ group compared to the DHT– group, although this was not statistically significant (p = 0.51). Finally, we observed that the average doses of BTX/A used in our patients were lower than in other studies. We can, therefore, hypothesize that CD patients with head tremor can benefit from being injected in more muscles at lower doses and this could be validated in future studies.

## Conclusion

Head tremor was seen in 57.6% of our CD patients and torticaput was the most common dystonic subtype associated with tremor. The majority of the patients with head tremor had a simple phenotype (one or two dystonic subtypes). The use of the “Col-Cap” classification could help to identify major muscles involved in tremor in CD patients. Injection guidance with USG documents that muscles are injected accurately; this tool may also improve the outcome and help to reduce the BTX dose by muscle. DHT+ patients possibly do not require higher botulinum toxin dosages than DHT– patients to get the effect of the treatment. Proper muscle selection is most important.
